# The fate of fertilizer nitrogen in a high nitrate accumulated agricultural soil

**DOI:** 10.1038/srep21539

**Published:** 2016-02-12

**Authors:** Zhi Quan, Bin Huang, Caiyan Lu, Yi Shi, Xin Chen, Haiyang Zhang, Yunting Fang

**Affiliations:** 1 Key Laboratory of Pollution Ecology and Environmental Engineering, Institute of Applied Ecology, Chinese Academy of Sciences, Shenyang 110016, P. R. China; 2 State Key Laboratory of Forest and Soil Ecology, Institute of Applied Ecology, Chinese Academy of Sciences, Shenyang 110164, P. R. China

## Abstract

Well-acclimatized nitrifiers in high-nitrate agricultural soils can quickly nitrify NH_4_^+^ into NO_3_^−^ subject to leaching and denitrifying loss. A 120-day incubation experiment was conducted using a greenhouse soil to explore the fates of applied fertilizer N entering into seven soil N pools and to examine if green manure (as ryegrass) co-application can increase immobilization of the applied N into relatively stable N pools and thereby reduce NO_3_^−^ accumulation and loss. We found that 87–92% of the applied ^15^N-labelled NH_4_^+^ was rapidly recovered as NO_3_^−^ since day 3 and only 2–4% as microbial biomass and soil organic matter (SOM), while ryegrass co-application significantly decreased its recovery as NO_3_^−^ but enhanced its recovery as SOM (17%) at the end of incubation. The trade-off relationship between ^15^N recoveries in microbial biomass and SOM indicated that ryegrass co-application stabilized newly immobilized N via initial microbial uptake and later breakdown. Nevertheless, ryegrass application didn’t decrease soil total NO_3_^−^ accumulation due to its own decay. Our results suggest that green manure co-application can increase immobilization of applied N into stable organic N via microbial turnover, but the quantity and quality of green manure should be well considered to reduce N release from itself.

Human-induced nitrogen (N) input increased over tenfold with respect to one century ago^1^,^2^. Excessive N fertilization caused accumulation of significant amounts of N beyond crop absorption in soils, largely in the form of nitrate (NO_3_^−^)^3^,^4^. Surplus NO_3_^−^ in soil is problematic because it is susceptible to loss by leaching or denitrification^2^,^5^, which is both economically and environmentally undesirable^4^,^6^. High nitrification potential and low NO_3_^−^ immobilization are responsible for NO_3_^−^ accumulation in agricultural soils^7^. Long-term N fertilization and tillage greatly increase the population and alter the community structure of ammonia oxidizing bacteria, as well as the nitrification capacity^4^,^8^,^9^,^10^. This is confirmed by the significant positively relationship between nitrification potential and soil NO_3_^−^ content^7^,^11^.

Co-application of N fertilizer with organic materials is especially common in high-input cropping systems, and it is considered to be a good agronomic practice as it is thought to elevate microbial bioavailability of the applied N and reduce N loss to the environment^12^. Much of immobilized N was speculated to be rapidly transformed to microbial residues or necromass as the average life cycle of N in microbial biomass was just several days^13^,^14^. While a recent study shows that soil newly synthesized amino acids are relatively easier to decompose in contrast with original soil amino acids^15^, the fate of newly immobilized N in various soil N pools is generally not well quantified.

In recent years, large areas of conventional cereal cultivation in China have been transferred to intensive greenhouse cultivation due to fast economic development and increased consumer demand^3^. Greenhouse soil cultivated with vegetables represents typical high NO_3_^−^ soil in agricultural system^3^,^16^. Constant monoculture, excessive fertilization, and high-intensity anthropogenic interference during greenhouse cultivation change the process of soil N transformation and accelerate the accumulation of NO_3_^− ^10,16. Thus, special attention is required to pay on N transformation in the NO_3_^−^ polluted soil (e.g., greenhouse soil) after the application of ammonium-based fertilizers.

Isotope ^15^N tracing technology is widely used to study the fate of chemical fertilizers and other N amendments in ecosystems. Previous studies on the fate of soil added ^15^N mainly focused on its transformation to extractable NH_4_^+^ and NO_3_^−^ pools, as well as N retention during a certain period (either short-term or long-term mostly)^7^,^9^,^17^,^18^. Due to the difficulties in experimental operation, dynamic and systematic studies on the fates of soil added ^15^N entering into various N pools (e.g. microbial biomass N, extractable organic N, mineral fixed N, non-extractable organic N) are rare, while these forms of N may play significant roles as important intermediates in biogeochemical N transformation processes in soil^15^,^19^.

Altering the fate of chemical fertilizer N to lessen NO_3_^−^ loss by manure co-application largely relies on the scale and the stability of manure-enhanced immobilization of fertilizer N. In this study, we conducted a 120-day incubation experiment to trace the fates of the inorganic fertilizer N alone or in combination with a green manure (as ryegrass in this study) to a greenhouse soil, using a ^15^N trace technique. The purpose of this study was to examine the effect of co-application of green manure on the fates of applied N entering into seven different N pools. Specifically, we examined if co-application of green manure promoted the incorporation of soil newly immobilized N into relatively stable organic N pools instead of remineralization, and in a long run, reduced NO_3_^−^ accumulation and loss risk in high-input greenhouse soil. We expected that green manure co-application would increase the stable immobilization of fertilizer N in the studied soil.

## Results

### Dynamics of soil extractable N (NH_4_^+^-N, NO_3_^−^-N, EON)

**Figure 1:**
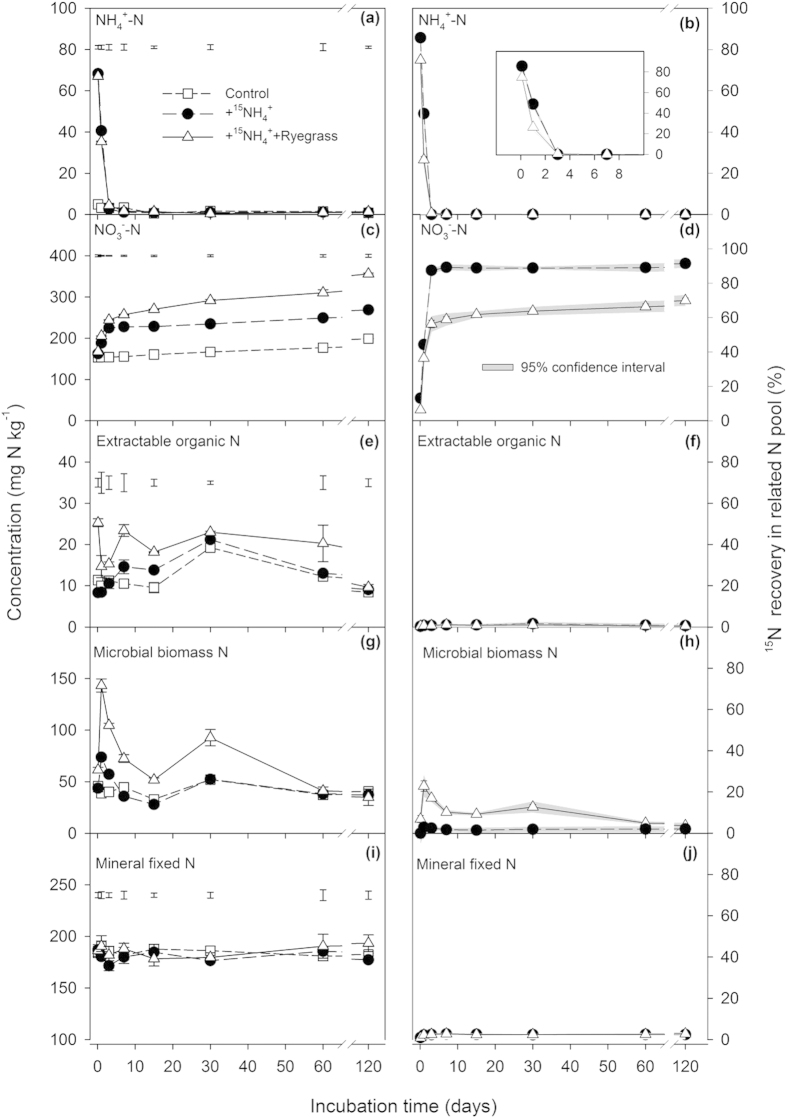
Concentrations (left) of extractable N speciation (NH_4_^+^-N, NO_3_^−^-N, EON), microbial biomass N (MBN) and mineral fixed N (MFN) and their ^15^N recoveries (right) after the addition of (^15^NH_4_)_2_SO_4_ alone (+^15^NH_4_^+^) or combined with ryegrass (+^15^NH_4_^+^+Ryegrass) to the tested soil. Error bars are smaller than the symbol when they are not visible. Separate bars in a line indicate the range of LSD (P = 0.05) for different treatments in the same incubation time. Confidence intervals (95%) of ^15^N recoveries in different pools were expressed as ±1.96 times of standard error.

In the control treatment, NH_4_^+^ concentration was 0.6 to 4.9 mg N kg^−1^, and did not change much over the 120-day incubation, while NO_3_^−^ concentration increased somewhat from 152 mg N kg^−1^ at the beginning of incubation to 199 mg N kg^−1^ at the end of incubation (Fig. 1a,c). In the N addition treatment, NH_4_^+^ decreased by 93–97% within 3 days from >80 mg N kg^−1^ to background level of 2.7–4.5 mg N kg^−1^. In contrast, NO_3_^−^ concentration increased rapidly to 225–244 mg N kg^−1^ from 152 mg N kg^−1^ in the first 3 days (Fig. 1b,d). From the 7 to 120 days, NO_3_^−^ concentration slightly increased, and ^15^N recovery in NO_3_^−^ pool, however, was relatively constant. Co-application of ryegrass significantly decreased the proportion of applied fertilizer NH_4_^+^ conversion to NO_3_^−^. During the days from day 3 to day 120, 56–70% of the applied fertilizer NH_4_^+^ was recovered as NO_3_^−^ in the +^15^NH_4_^+^+Ryegrass treatment, being significantly lower than that (87–92%) in the +^15^NH_4_^+^ treatment (Fig. 1d).

Extractable organic N (EON) was 8.4 to 25.3 mg N kg^−1^ and accounted for 3–11% of total extractable nitrogen (TEN, NH_4_^+^-N + NO_3_^−^-N + EON) (Fig. 1e). Ryegrass addition slightly increased EON concentrations. Less than 2% of the applied fertilizer NH_4_^+^ was recovered as EON, and there was no significant difference in the ^15^N recovery in EON between the two ^15^NH_4_^+^ addition treatments (Fig. 1f).

### Dynamics of soil microbial biomass N (MBN) and mineral fixed N (MFN)

In the control treatment, MBN concentrations was relatively constant, being 32.9 to 52.0 mg N kg^−1^ (Fig. 1g). NH_4_^+^ addition increased MBN concentration slightly only in the first 3 day of incubation. However, co-application of ryegrass with NH_4_^+^ increased MBN significantly in the first 30 days. After 60 days, there was no significant difference in MBN concentrations among the three treatments. In the +^15^NH_4_^+^ treatment, 1.5–2.9% of the applied fertilizer NH_4_^+^ was recovered to microbial biomass (Fig. 1h). In the +^15^NH_4_^+^+Ryegrass treatment, the proportion to microbial biomass changed over the incubation, from 5% in the 0.1 day increased to a maximum of 22% in the 1 day and decreased to 4% at the end of incubation.

MFN concentrations were comparable to NO_3_^−^ concentration, but were not different among the three treatments (Fig. 1i). The ^15^N recovery in MFN pool did not change and stabilize at 2–3% in the two N addition treatments during day 1–120 (Fig. 1j).

### Incorporation into soil organic matter (SOM) and N loss

**Figure 2:**
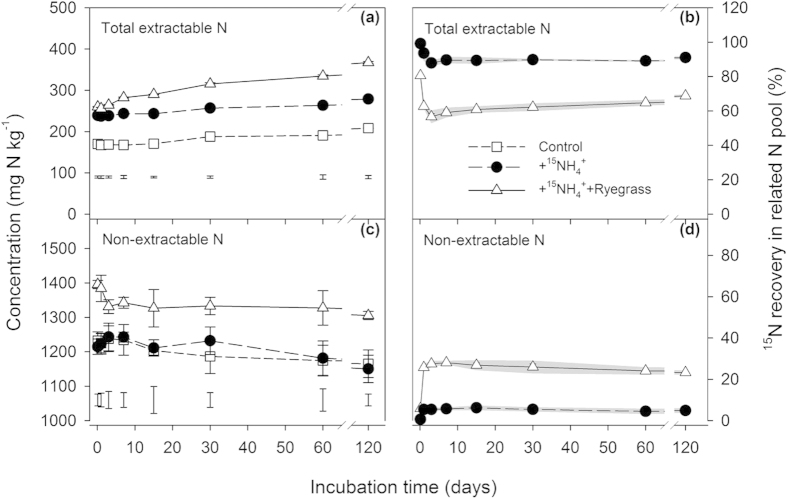
Concentrations (left) of total extractable N (TEN, = NH_4_^+^-N + NO_3_^−^-N + EON) and non-extractable N (NEN) and their ^15^N recoveries (right) after the addition of (^15^NH_4_)_2_SO_4_ alone (+^15^NH_4_^+^) or combined with ryegrass (+^15^NH_4_^+^+Ryegrass) to the tested soil. Error bars are smaller than the symbol when they are not visible. Separate bars in a line indicate the range of LSD (P = 0.05) for different treatments in the same incubation time.

The two ^15^N addition treatments yielded different ^15^N recoveries in TEN and non-extractable N (NEN) pools (Fig. 2b,d). The ^15^N recoveries in TEN pool were 88–99% for the +^15^NH_4_^+^ treatment and 57–81% for the +^15^NH_4_^+^+Ryegrass treatment during the 120-day incubation, and showed a slight increase after an initial sharp drop. A reverse trend was found for the ^15^N recovery in NEN pool (Fig. 2).

**Figure 3:**
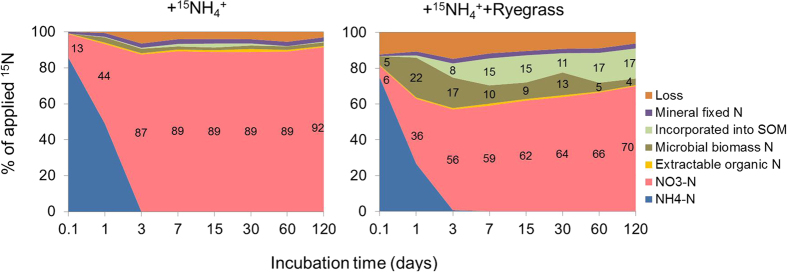
Changes in recoveries of ^15^N in the soil as NH_4_^+^-N, NO_3_^−^-N, extractable organic N (EON), microbial biomass N, incorporated into SOM, mineral fixed N fractions and loss during the 120-day lab incubation after the addition of (^15^NH_4_)_2_SO_4_ alone (+^15^NH_4_^+^) or combined with ryegrass (+^15^NH_4_^+^+Ryegrass) to the tested soil. ^15^N recovery in SOM pool was calculated as the difference of recoveries in “non-extractable N” and “microbial biomass N + mineral fixed N”. Nitrogen loss is the unrecovered ^15^N neither in extractable nor non-extractable pools. Numbers in the areas are the corresponding ^15^N recoveries in relevant N pool at the related incubation time.

We defined the ^15^N recovery in NEN pool minus in MFN and MBN pools (NEN-MFN-MBN) in the unfumigated post-extracted soil as incorporation into SOM, and defined the unrecovered ^15^N (neither in TEN nor NEN pools) as loss (Fig. 3). During day 3–120 in the +^15^NH_4_^+^ treatment, only 0.1–2% and 3–6% of the applied fertilizer N was incorporated into SOM and lost, respectively. When ryegrass was co-applied, 8–17% of the applied fertilizer N was incorporated into SOM and 6–15% of applied N was lost, respectively (Fig. 3).

## Discussion

### Nitrification

**Figure 4:**
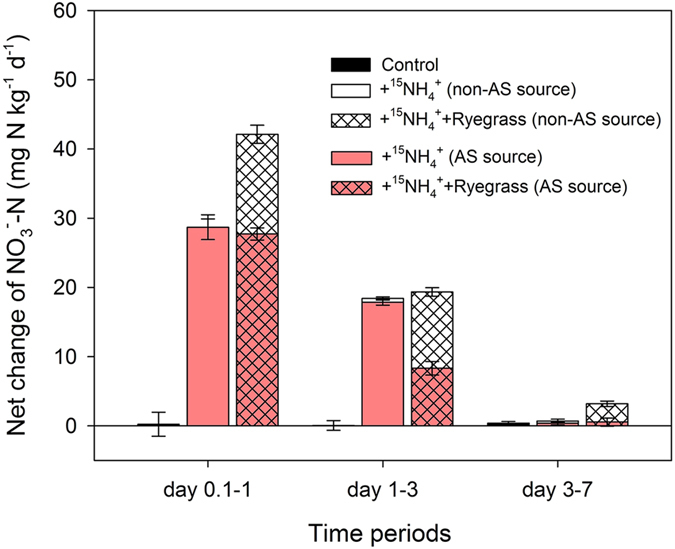
Net change in NO_3_^−^-N concentrations during day 0.1–1, day 1–3, and day 3–7 after the addition of (^15^NH_4_)_2_SO_4_ alone (+^15^NH_4_^+^) or combined with ryegrass (+^15^NH_4_^+^+Ryegrass) to the tested soil. AS: ammonium sulfate.

Our results show that the studied greenhouse soil is characterized by high NO_3_^−^ accumulation (>150 mg N kg^−1^) and very fast nitrification. The net nitrification rates (net NO_3_^−^ change) of the applied fertilizer NH_4_^+^ were 28.7 and 27.7 mg N kg^−1^ d^−1^ for the +^15^NH_4_^+^ and +^15^NH_4_^+^+Ryegrass treatments respectively during day 0.1–1, and 17.9 and 8.3 mg N kg^−1^ d^−1^ during day 1–3 (Fig. 4), much higher than the reported values for many other ecosystems^4^,^11^,^20^. Nitrifiers were acclimatized with high quantity and improved function in the tested soils due to high-rate fertilization and long-term vegetable cultivation but limited C input^20^,^21^. In contrast, in N-limited forest soil, low N input and sufficient available C to fuel high denitrification and immobilization, prevents the NO_3_^−^ accumulation^7^,^22^,^23^.

The relative strength of N mineralization and immobilization mainly depends on the C/N of added organic material as well as the incubated soil^24^,^25^,^26^. The total rate of nitrification (including ^14^N and ^15^N) in the ryegrass co-applied soil was not reduced in this study (Figs 1c and 4), primarily due to the supply of non-labelled NH_4_^+^ from relatively fast mineralization of the ryegrass with a C/N ratio only 12.4 (soil C/N ratios were 10.5, 10.0 and 10.2 respectively after the addition for Control, +^15^NH_4_^+^ and +^15^NH_4_^+^+Ryegrass treatments). The total nitrification rate and N release would get reduced if organic material with a higher C/N than ryegrass was applied^27^. But the use of organic material with a wider C/N ratio (e.g. crop straw) may not really favor transformation of NH_4_^+^ into organic N pool instead of NO_3_^−^ pool, due to the desynchrony between slow release of available C from crop straw and fast nitrification of applied NH_4_^+^ in greenhouse soil. Therefore, reducing nitrification of applied fertilizer N by co-application of organic material in high-input agricultural soils should well consider the quality (e.g. C/N, carbon availability) of added organic material^22^,^26^,^27^.

### Incorporation into MBN and SOM

Our study dynamically evaluated the stability and further transformation of microbially assimilated N using the indicator “non-extractable organic ^15^N”. The results suggest that co-application of green manure led to effective NH_4_^+^ immobilization and stabilization in soil (Fig. 3). During day 1–120, 23–28% of the applied fertilizer N was incorporated into non-extractable organic N, initially primarily as MBN, and eventually primarily as SOM. According to the low soil NH_4_^+^ concentration after day 3, and the low microbial assimilation to NO_3_^−^ in agricultural soils^7^,^28^, we propose that the slow decline of ^15^N recovery in non-extractable organic N pool (^15^N incorporated into MBN and SOM) after day 3 was dominated by the remineralization of newly immobilized organic ^15^N (Fig. 3). Our results also show that newly immobilized organic ^15^N is a relatively stable N pool that can accumulate rapidly but is not readily accessible to microbial mineralization.

Studies of soil organic matter after ^15^NH_4_^+^ or ^15^N-urea addition by nuclear magnetic resonance technique (NMR) confirm that the immobilized organic ^15^N is concentrated as the form of peptide or amide^17^,^29^,^30^, which is regarded as a microbial source^19^,^31^. In this study, the ryegrass-induced rapid up-down change of ^15^N recovery in MBN (Fig. 1h), as well as the trade-off changes of ^15^N recoveries in MBN and “incorporated into SOM” pools (Fig. 3), indicate that the initial immobilization of ^15^NH_4_^+^ and its subsequent transformation in the soil should be mainly microbially mediated^32^,^33^,^34^, and the disappeared MB^15^N was mainly further incorporated into SOM, a stable soil organic N pool that hard to decompose^35^. Appel *et al*.^36^ confirmed that almost no extractable ^15^N was extracted from soils when ^15^N-labelled bacterial biomass was added prior to extraction. This goes against usual understanding that the newly immobilized N will release to bioavailable N after the death and breakdown of live microbes. It is expected that the soil N immobilized by microbes may contribute a lot to the low but long availability of residual fertilizer-N (residual effect) to subsequent crops^37^,^38^.

The biotic immobilization of added NH_4_^+^ was mainly initiated and regulated by active microorganisms^39^. MB^15^N was too variable within a short period^26^ as also evidenced by this study to reliably reflect the scale of ^15^NH_4_^+^ immobilization, especially in a relatively long period. In contrast, newly immobilized organic ^15^N, composed of ^15^N incorporation into both MBN and SOM, can be fast accumulated but slowly mineralized in the long-term process (Fig. 3), and may be a better indicator versus MB^15^N to more reliably quantify the microbial assimilation of added ^15^NH_4_^+^ in a long period.

Immobilization of N in soils might also be a result of abiotic reaction^40^,^41^, but the proportion is generally low in agricultural soil^42^ and in our study soil (Fig. 1j). Nevertheless, no matter how much the immobilization of applied ^15^NH_4_^+^ is biotically or abiotically regulated, the evidence provided by the present study suggested that applying green manures (e.g. ryegrass) to soil could significantly enhance the stable immobilization of applied NH_4_^+^. This is very significant for N management in intensive cultivation systems (e.g. greenhouse cultivation, orchard, vineyard, etc.), where large amounts of N fertilizer are applied.

### Incorporation into EON, MFN and N loss

Compared to NO_3_^−^ and MBN, the proportions of ^15^N entering into EON and MFN pools were low in this study (Figs 1 and 3), which was consistent with previous studies^26^,^43^,^44^. Because most of EON in soils is resistant to decomposition, rapid turnover of easily degradable EON released from fresh organic matter will lead to the low contribution of added organic material to the total EON pool^44^. With the decline of the recovery of MB^15^N, the EO^15^N recovery kept continuously low (Fig. 3), reflecting indirectly a low contribution of the disappeared MBN to EON during the 120-day incubation. The content of clay-fixed ammonium depends on the degree of K saturation of the interlayers of 2:1 clay minerals^45^,^46^. Low ^15^N recovery in MFN pool in this study may be due to the block of sufficient K^+^ and NH_4_^+^ resulting from high rate fertilization of K and N in greenhouse vegetable cultivation process. Available K in the greenhouse soil is up to 201.7 ± 2.8 mg kg^−1^, being much higher than that in nearby cropland soil (74.4–127.0 mg kg^−1^)^35^,^47^ Still, low moisture change during the first three days of the incubation may also contribute a part to the low fixation as soil dry-wet cycle is also thought to be an important factor controlling NH_4_^+^ fixation^45^.

Co-application of ryegrass might increase the loss of applied fertilizer NH_4_^+^ (Fig. 3), in the forms of nitrogenous gases (NH_3_, N_2_, N_2_O, NO, HONO, etc.). The quantity of loss via ammonia volatilization in the soil was low (measured by absorption with dilute H_2_SO_4_, Kjeldahl distillation and titration) and the difference was not significant among treatments (<0.5 mg N kg^−1^ during the 120-day incubation period, detailed data was not shown here). Compared to the treatment with only NH_4_^+^ addition, we suggest that denitrification was fueled by available C from ryegrass and thereby increased the loss of gaseous N. Also, fast ryegrass mineralization rapidly stimulated aerobic microbial activity, likely reducing the permeability and availability of oxygen in soil pores to increase denitrifying loss of ^15^NO_3_^−^ in the very initial period, which was verified by the observation of lower water perviousness (need more time to completely infiltrate) in the soil with ryegrass addition than that without ryegrass addition when we added deionized water at regular intervals.

## Methods

### Soil and ryegrass samples preparation

In the present study, Fluvo-aquic soil (silty loam, *Hapli-Udic Cambisols* in Chinese soil taxonomy) samples were collected from the surface layer (0–20 cm) of a greenhouse field planted with pepper (*Capsicum annuum L.*) in Damintun town, Xinmin county, Liaoning province (122°50´E, 41°59´N). The greenhouse soil had a 7-yr history of vegetable cropping (cucumber, tomato, pepper, etc.), and before that it was under maize cultivation for decades. The soil had a low C/N ratio of 10.5, and a high NO_3_^−^ accumulation (152 mg N kg^−1^, Table 1).

In this study, ryegrass (*Lolium multiflorum* Lam.) samples was applied as a green manure in the greenhouse soil, which was obtained by culturing ryegrass in pots (21 cm in diameter and 20 cm in height filled with 3.5 kg soil) until it matured. Each pot was applied with 1 g of non-labelled urea.

### Experimental design

Three treatments were set with 4 replicates for each treatment: (1) Control, soil without additions; (2) +^15^NH_4_^+^, adding as ^15^N-labelled ammonium sulfate ((NH_4_)_2_SO_4_); (3) +^15^NH_4_^+^ + Ryegrass, adding as ^15^N-labelled (NH_4_)_2_SO_4_ and non-labelled ryegrass. ^15^N abundance of (NH_4_)_2_SO_4_ was 50.17 atom%, and (NH_4_)_2_SO_4_ was added at a rate of 80 mg N kg^−1^ dry soil, being equivalent to the addition of 200 kg N ha^−1^ into the 0–20 cm soil layer in the field. The ryegrass was smashed to powder and sieved through 0.5 mm before use, and added at a rate of 4.21 g kg^−1^ dry soil. Selected properties of the soil and ryegrass were listed in Table 1.

For the laboratory incubation, soil was sieved through 2 mm and mixed homogeneously after gravimetric soil moisture was lowered to approximately 15%, and then pre-incubated at 25 °C for 7 days. Thereafter, 25 g soil (on an oven-dried basis) was weighed into polyethylene plastic centrifuge cups (60 mm in diameter and 81 mm high, n = 64 for each treatment). After mixing the soil thoroughly with ryegrass for the +^15^NH_4_^+^+Ryegrass treatment, aqueous solution of (^15^NH_4_)_2_SO_4_ or deionized water was added over the soil surface with a pipette to bring the gravimetric soil moisture uniformly to 25.3% (corresponded to 60% water filled pore space). The application was made drop-wise to the soil in the cup and the soil then mixed to ensure the application was even. Finally, the soil was mechanically compressed to field bulk density (1.25 g cm^−3^) using a small disc with a handle. All cups were covered with parafilm punctured with a needle to maintain aerobic conditions and incubated in an automatically controlled incubator (25 °C). Distilled H_2_O was added at regular intervals (4 days) to maintain the soil moisture and prevent from decreasing the microbial activity due to water limitation during the 120-day incubation.

### Sample processing and chemical analysis

**Figure 5:**
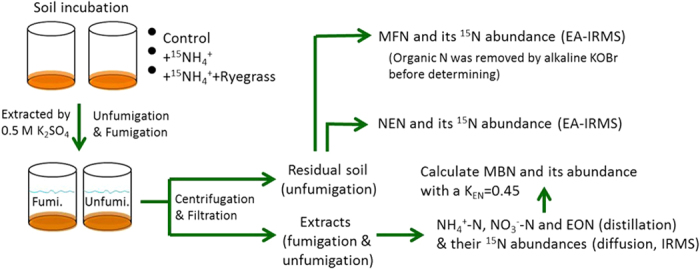
Experimental procedures to determine concentrations and abundances of the added ^15^N of extractable N components (ammonium, nitrate, organic-N), microbial biomass N, non-extractable N and mineral fixed N in tested soils.

Approximately 0.1 (2.5 h), 1, 3, 7, 15, 30, 60 and 120 days after adding (^15^NH_4_)_2_SO_4_, eight replicates of each treatment were destructively sampled, extracted directly (four cups) or after fumigation (four cups) with 100 ml 0.5 mol l^−1^ K_2_SO_4_ for 30 minute on a shaker (220 rev min^−1^) (Fig. 5). The fumigation was carried out with alcohol-free chloroform in a desiccator lined with wet filter paper at 25 °C for 24 h in the dark^48^. After centrifugation (5000×g, 10 min), the supernatant was filtered through cellulose acetate membranes (0.45 μm, Xinya, Shanghai) that had been prewashed with 0.5 mol l^−1^ K_2_SO_4_. The extracts were determined immediately or stored at −20 °C until future analysis. The soil residue was extracted two more times with K_2_SO_4_ solution (100 ml each, supernatant was discarded) to remove extractable N thoroughly from soil particles. All residual soil was carefully recovered, air-dried, ground, and sieved (<0.15 mm).

Soil extracts (20 ml) were distilled after the addition of MgO and Devarda’s alloy respectively for quantifying the content of NH_4_^+^-N and NO_3_^−^-N^49^. A modified micro-diffusion procedure using Teflon strips was performed to prepare samples for analysis of ^15^N abundance in the NH_4_^+^ and NO_3_^−^ pools^50^. After distillation above, the remaining samples were digested with H_2_SO_4_ and H_2_O_2_ to convert all organic N to NH_4_^+^, and subsequently distilled with excess NaOH solution to determine extractable organic N (EON) concentration. Similarly, after conversion of EON (after diffusion above) to NH_4_^+^, the digested solution was transferred to plastic container (resistant to acid and alkaline solutions), kept frozen at −20 °C overnight, and then diffused with excess NaOH solution (4 °C) to prepare samples for analysis of ^15^N abundance in the EON pool^51^. The ^15^N abundances in the prepared samples were obtained by an elemental analyzer (Flash EA1112, Thermo Finnigan, USA) coupled with an Isotope-Ratio Mass Spectrometer (Delta plus XP, Thermo Finnigan, USA) (EA-IRMS). The standard deviation of ^15^N abundance measurements (δ^15^N) for the standard sample is less than 0.3‰ (8 replicates).

Concentrations of non-extractable N (NEN) and mineral fixed NH_4_^+^-N (MFN), and their ^15^N abundances in residual soil after extraction mentioned above were determined by the EA-IRMS. Organic N in residual soil was removed by excessive fresh prepared alkaline KOBr solution before determining MFN^52^,^53^. Soil and ryegrass total organic carbon (TOC) was measured using the solid module of TOC/TN analyzer (Multi N/C 3100, Analytikjena, Germany). Soil and ryegrass total N were determined by the Kjeldahl method. Soil available potassium (K) was determined by a flame photometer (6400A, Shanghai) after extraction with 1 mol l^−1^ ammonium acetate (v/w = 10:1).

### Calculation

The soil microbial biomass nitrogen (MBN) was quantified using the following formula with a K_EN_ = 0.45 54.


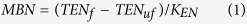


The ^15^N recovery in each N pool was calculated based on the simple mass balance and a mixing model in each pool, as follows^41^:


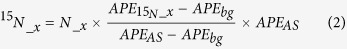



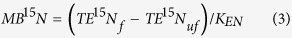



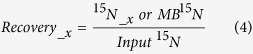


where, TEN (total extractable N) = NH_4_^+^-N + NO_3_^−^-N + EON. Letters of “f” and “uf” respectively mean “fumigated” and “unfumigated”; *N*_*_x*_ is the amount of soil N components (NH_4_^+^-N, NO_3_^−^-N, EON, MFN or NEN, mg kg^−1^); ^15^*N*__*x*_ is the amount of ^15^N (mg kg^−1^) from labelled ammonium sulfate added in related N pool; 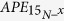, 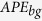 and 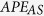 are the atom percent excesses (%) of N component in related N pool, background and ammonium sulfate added, respectively; *Recovery*_*x*_ is the percentage of the ^15^N tracer recovered in the labelled N pool; *Input*^15^*N* is the amount of ^15^N in ammonium sulfate added (mg kg^−1^).

Net nitrification rates were calculated as the difference between final and initial NO_3_^−^-N concentrations for three time intervals, days 0.1–1, days 1–3 and days 3–7 .

### Statistical analyses

Data are expressed on oven-dried soil basis, and subjected to analyses of variance using SPSS for Windows v16.0 software package (SPSS Inc., Chicago, IL, USA). All figures were obtained from SigmaPlot 12.5. Error bars in figures represented standard errors. The differences between means of variables for different treatments were statistically tested by ANOVA procedure with least significant difference (LSD) test. The standard errors of composite variables (the concentration of MBN; the recoveries of ^15^NH_4_^+^, ^15^NO_3_^−^, EO^15^N, MB^15^N, MF^15^N, TE^15^N, NE^15^N, and the net change of NO_3_^−^) were calculated through the formula of Gaussian error propagation^55^. Confidence intervals (95%) of ^15^N recoveries in different pools were expressed as ±1.96 times of standard error.

## Additional Information

**How to cite this article**: Quan, Z. *et al*. The fate of fertilizer nitrogen in a high nitrate accumulated agricultural soil. *Sci. Rep.* **6**, 21539; doi: 10.1038/srep21539 (2016).

## Figures and Tables

**Table 1:** Chemical properties of the tested soil and ryegrass.

	Soil	Ryegrass
pH^ξ^	6.41	–
Total organic C (TOC, g kg^−1^)	14.9	440
Total N (TN, g kg^−1^)	1.41	35.5
TOC/TN	10.5	12.4
Extractable organic C (g kg^−1^)^§^	0.13	77.7
Extractable NO_3_^−^-N (mg kg^−1^)	152	1917
Extractable NH_4_^+^-N (mg kg^−1^)	5	593
Extractable organic N (mg kg^−1^)	8	7760
Extractable N/TN (%)	11.7	28.9
Available K (mg kg^−1^)	201.7	–

^*ξ*^1:2.5 (soil: deionized water). ^§^Extractable C and N were extracted by 2 mol l^−1^ KCl (1:5 for soil and 1:20 for ryegrass, w/v).
